# Relationship between daily isoflavone intake and sleep in Japanese adults: a cross-sectional study

**DOI:** 10.1186/s12937-015-0117-x

**Published:** 2015-12-29

**Authors:** Yufei Cui, Kaijun Niu, Cong Huang, Haruki Momma, Lei Guan, Yoritoshi Kobayashi, Hui Guo, Masahiko Chujo, Atsushi Otomo, Ryoichi Nagatomi

**Affiliations:** 1 Division of Biomedical Engineering for Health & Welfare, Tohoku University Graduate School of Biomedical Engineering, Sendai, Japan; 2 Department of Epidemiology, School of Public Health, Tianjin Medical University, Heping District, Tianjin, People's Republic of China; 3 Department of Medicine and Science in Sports and Exercise, Tohoku University Graduate School of Medicine, Sendai, Japan; 4 Tianjin University of Sport, Tianjin, China

**Keywords:** Cross-sectional study, Isoflavone, Sleep duration, Sleep quality, Estrogen, Japanese adults

## Abstract

**Background:**

Isoflavones comprise a class of phytoestrogens that resemble human estrogen in chemical structure, and have weak estrogenic effects. Because estrogen modulates sleep duration and quality, we hypothesized that isoflavones would have a beneficial effect on sleep status in a way similar to estrogen. We conducted a cross-sectional study to investigate the relationship between daily isoflavone intake and sleep status in Japanese subjects.

**Methods:**

Our study included 1076 Japanese adults aged 20-78 years. Daily isoflavone intake was assessed using a brief self-administered diet history questionnaire, and sleep was evaluated using a self-reported questionnaire.

**Results:**

The prevalence of regular sleep duration (7–8 h/day) and sufficient sleep quality were 13.3 % and 56.2 %, respectively. After adjusting for potential confounding factors, the odds ratios (95 % CIs) for optimal sleep duration (7–8 h) when higher isoflavone intakes (Q2–Q4) were compared with low isoflavone intake (Q1) were Q2: 0.94 (0.53–1.56); Q3: 1.28 (0.73–2.24); and Q4: 1.84 (1.06–3.18) (*p* for trend = 0.013). In the final adjusted model, sufficient sleep quality across categories of isoflavone intake was Q1: 1.00 (reference); Q2: 1.30 (0.91–1.84); Q3: 1.48 (1.03–2.12); and Q4: 1.78 (1.22–2.60); (*p* for trend = 0.002).

**Conclusion:**

Higher daily isoflavone intake was positively associated with optimal sleep duration and quality in a Japanese population. This finding suggests that daily isoflavone intake may have a potentially beneficial effect on sleep status.

## Introduction

Sleep is a homeostatic process that involves an active and periodic biological state that is crucial to good physical and mental health [1]. Sleep also has beneficial effects on stress reactions and circadian rhythms [2]. However, sleep disorders have increasingly become a major focus of public health [3], and complaints concerning sleep problems and symptoms of insomnia are common in older adults [4]. A large prospective cohort study demonstrated that sleep quality and sleep length (both short and prolonged sleep) are related to increased mortality; in addition, risk of death was lowest in those with an average of 7–8 h of sleep at night [5]. Many studies have indicated that sleep complaints (e.g., difficulties falling asleep) are associated with coronary artery disease mortality in men [6]; furthermore, 7–8 h of sleep per night can reduce the risks of coronary heart disease [7, 8], hypertension [9], diabetes [10], and obesity [11].

In recent years, the understanding of the effects of estrogen on brain function has increased considerably. Estrogen acts on the brain primarily via the same neurotransmitters that are involved in sleep regulation [12]. The majority of research on hormone replacement therapy focuses on the role of estrogen and its positive effects on sleep. Compared with a placebo, estrogen replacement therapy has been shown to alleviate insomnia [13]; other reported benefits include improvements in falling asleep, diminished nocturnal restlessness, and fewer awakenings [14]. Isoflavones, a class of phytoestrogens, share structural similarities with the mammalian hormone 17-β estradiol and have weak estrogenic effects. Isoflavones are relatively abundant in the circulation of people and animals that consume soy. Many studies have shown that isoflavones can bind to and mediate transcription through estrogen receptors α and β [15–17]. Several studies have also shown that in the absence of estrogen, isoflavones have weak estrogenic effects, while in the presence of estrogen, they may exert an antagonistic effect [18, 19].

Therefore, it is conceivable that isoflavones may have potentially beneficial effects on sleep status. To our knowledge, only two studies have examined the relationship between isoflavones and sleep. Both determined that isoflavone treatment was effective on sleep disorders in postmenopausal women [20, 21]. No studies, however, have investigated the relationship between daily intake of isoflavone from food and sleep status in the general population. Here, we conducted a cross-sectional study to investigate the relationship between daily isoflavone intake and sleep duration and quality in a Japanese population.

## Methods

### Study population

We used data from a prospective cohort study conducted to investigate the risk factors of chronic diseases among adult employees. The study was based on annual health examinations at the Sendai Oroshisho Center [22]. To stratify for potential confounding variables, we added several assessment parameters to the health examination: 1) questionnaires (details provided below), 2) physical performance measurements (e.g., leg extension power and grip strength), 3) blood examination (e.g., adiponectin), and 4) daily physical activity (PA) assessment using a 3-dimensional accelerometer [23].

**Fig. 1:**
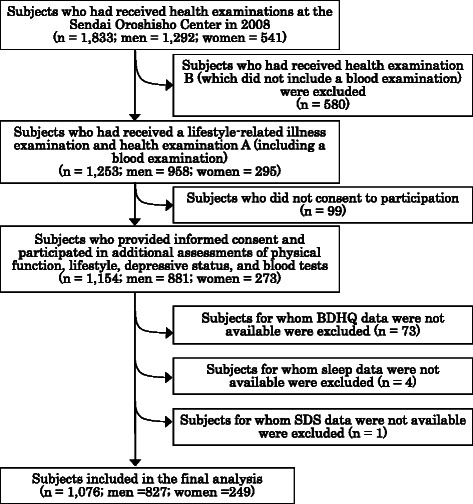
Flow chart of the sample selection process

The sample selection process is described in Fig. 1. In total, 1833 individuals had previously undergone one of two health examinations: lifestyle-related illnesses and health examination A, which included blood examinations, or health examination B, which did not include blood examinations [22]. We invited all subjects who had undergone the lifestyle-related illnesses and health examination A to participate in the study (n = 1253). Of those invited, 1154 agreed to participate and provided informed consent for their data to be analyzed (response rate 92.1 %). The Institutional Review Board of the Tohoku University Graduate School of Medicine approved the protocol for our study.

We excluded subjects without brief self-administered diet history questionnaire (BDHQ) (n = 73), sleep (n = 4), or Self-Rating Depression Scale (SDS) (n = 1) data. Therefore, the final study population comprised 1076 subjects (827 men and 249 women).

### Assessment of dietary intake

Habitual dietary intake was assessed using the BDHQ that included questions concerning the monthly food intake frequency of 75 food items, along with their specified serving sizes [24].

Isoflavone intake consisted of daidzein and genistein, the major isoflavones found in soybeans. Typical isoflavone intake was measured using 3 soy foods that are rich in isoflavones: natto, tofu, and fried tofu. The participants indicated the mean frequency of consumption of these foods over the past month by checking 1 of 7 frequency categories, ranging from “almost never” to “2 or more times/day.” The mean daily consumption of nutrients was calculated using an ad hoc computer program developed to analyze the questionnaire. The Japanese food composition tables (5th edition) were used as the nutrient database. Energy-adjusted values by the density method (/1000 kcal) were used for all nutrients. We summarized the quantity of daily isoflavone intake in quartiles as follows, Q1: 0–10.96 mg/1000 kcal/day; Q2: 10.97–17.99 mg/1000 kcal/day; Q3: 18.00–26.73 mg/1000 kcal/day; and Q4: 26.74–83.06 mg/1000 kcal/day.

### Assessment of sleep

We assessed sleep using a standard questionnaire that included sleep-related questions. Information on sleep duration was obtained by asking, “How many hours do you usually sleep per day?” Six response alternatives were provided: < 5 h, 5–6 h, 6–7 h, 7–8 h, 8–9 h, and ≥ 9 h. Sleep duration was then categorized into 2 classes: 7–8 h; and < 7 h or ≥ 8 h. Information on sleep quality was obtained by asking “Do you usually feel refreshed after sleep?” Possible answers were “yes” and “no.” The use of hypnotics was assessed in the question “Have you used hypnotic drugs in the past month?” Possible answers were “yes” and “no.”

### Assessment of other variables

Depressive symptoms were assessed according to the Japanese version of the SDS [25]. An SDS score ≥ 50 was defined as the cut-off point indicating relatively mild or severe depressive symptoms [26]. Blood pressure was measured twice, on the upper left arm, with an automatic device (YAMASU605P; Kenzmedico Co., Ltd., Saitama, Japan) after 5 min of rest in the sitting position. The mean of the 2 measurements was used for analyses.

Blood samples were collected in siliconized vacuum glass tubes containing sodium fluoride. Fasting blood glucose concentrations were measured using enzymatic methods (Eerotec Co., Ltd., Tokyo, Japan). The concentrations of triglycerides, low-density lipoprotein cholesterol, and high-density lipoprotein cholesterol were measured using enzymatic methods with the appropriate kits (Sekisui Medical Co., Ltd., Tokyo, Japan).

Body mass index (BMI) was calculated as weight/height^2^ (kg/m^2^). The criteria from American Heart Association scientific statements were used to define metabolic syndrome. Participants were considered to have metabolic syndrome if they presented with ≥ 3 of the following risk factors: (1) abdominal obesity: waist circumference ≥ 90 cm (for men) or ≥ 80 cm (for women); (2) hypertension: systolic BP ≥ 130 mmHg or diastolic BP ≥85 mmHg, or anti-hypertensive drug treatment; (3) elevated triglycerides: serum triglycerides ≥ 150 mg/dL or drug treatment for elevated triglycerides; (4) reduced HDL-C: HDL-C < 40 mg/dL (for men) or < 50 mg/dL (for women), or the use of medications for reduced HDL-C; and (5) hyperglycemia: fasting plasma glucose ≥ 100 mg/dL or drug treatment for elevated glucose. Level of education was determined as the last qualification attained, and was subsequently divided into 2 categories: < college or ≥ college. Subjects were asked whether they had a history of physical illness or if they were currently taking any medication; answers were given as “yes” or “no.” Information on age, sex, smoking status, and occupation was obtained using a questionnaire survey. Daily PA was determined using the International Physical Activity Questionnaire (IPAQ), and total daily PA was calculated as follows: METs × h/week [27]. PA was then divided into two categories, high and low, matched for number of individuals.

### Statistical analysis

Descriptive data are presented as the means (95 % CIs) or as percentages. Sleep duration and sleep quality were used as dependent variables and isoflavone intake categories (Q1–Q4) were used as independent variables. The differences in variables between the isoflavone intake categories were examined using analysis of variance for continuous variables or the *χ*^2^ test for variables of proportion. Multivariate logistic analysis was used to determine the relationships between isoflavone intake categories and sleep duration and quality, and to adjust for potentially confounding variables. Model 1 was adjusted for age, sex, and BMI. Model 2 was adjusted for those variables in Model 1, along with intake of vitamins B12, C, and D, and coffee consumption. Model 3 was adjusted for those variables in Model 2, along with smoking and drinking habits, PA, level of education, SDS score, metabolic syndrome, high-sensitivity C-reactive protein (hsCRP), occupation, and hypnotic drug use. The final multivariate logistic analysis was performed with the forced entry of all factors considered as potential covariates. All *p*-values for linear trends were calculated using the isoflavone intake categories (Q1, Q2, Q3, Q4). The interactions between isoflavone intake and all confounding variables for sleep duration and sleep quality were tested through the addition of the cross-product terms to the regression model. All statistical analyses were performed using the SPSS/PC statistical software version 17.0 for Windows (SPSS, Inc., Chicago, IL); the significance level was set at *p* < 0.05.

## Results

Data were obtained from 1076 subjects; 143 (13.3 %) subjects were classified as having normal sleep duration (7–8 h), and 605 (56.2 %) subjects were classified as sufficient sleepers with regard to sleep quality. Baseline characteristics, according to isoflavone intake categories, are presented in Table 1. The proportions of subjects who were ex-smokers or nonsmokers, had high PA, were elderly, and were significantly high across the isoflavone intake quartiles (*p* for trend *p* < 0.001; variables: *p* < 0.048, *p* < 0.001, and *p* = 0.016, respectively). The mean dietary intakes for all items, except coffee consumption, was significantly high across the categories of isoflavone intake (*p* for trend < 0.001). The mean coffee consumption and proportions of subjects who were current smokers were significantly lower across categories of isoflavone intake (*p* for trend = 0.028; variable: *p* < 0.001).

**Table 1:** Subject characteristics according to categories of daily isoflavone intake

	Categories of isoflavone intake^a^				*p* for trend^b^
	Q1 (Low)	Q2	Q3	Q4 (High)	
n	269	269	269	269	
Sex (male; %)	75.8	79.6	77.3	74.7	0.628
Age (years)	43.3 (42.0, 44.5)^c^	45.5 (44.3, 46.7)	46.9 (45.7, 48.2)	49.6 (48.4, 50.9)	<0.001
BMI (kg/m^2^)	23.1 (22.7, 23.6)	23.2 (22.7, 23.6)	23.5 (23.1, 24.0)	22.9 (22.5, 23.3)	0.672
Dietary intake					
Vitamin B12 (μg/1000 kcal)	4.0 (3.7, 4.3)	4.6 (4.3, 4.9)	4.9 (4.6, 5.2)	4.9 (4.6, 5.2)	<0.001
Vitamin C (mg/1000 kcal)	42.3 (39.4, 45.1)	48.1 (45.2, 50.9)	52.9 (50.0, 55.7)	60.2 (57.3, 63.0)	<0.001
Vitamin D (mg/1000 kcal)	5.0 (4.6, 5.5)	5.9 (5.4, 6.4)	6.8 (6.3, 7.3)	7.1 (6.6, 7.6)	<0.001
Coffee consumption (g/d)	254.6 (231.0, 278.2)	236.7 (213.1, 260.3)	247.7 (224.1, 271.3)	211.4 (187.7, 235.0)	0.028
PA (high; %)	47.6	48.0	45.0	59.5	0.016
Smoking status (%)					
Current smoker	55.3	46.9	42.3	36.1	<0.001
Ex-smoker	8.2	12.2	15.0	14.5	0.016
Nonsmoker	34.6	39.8	45.7	51.3	<0.001
Drinking status (%)					
Drinking everyday	28.3	30.5	26.0	25.3	0.272
Drinking occasionally	48.0	49.8	49.8	53.5	0.220
Nondrinker	23.8	19.7	24.2	21.2	0.768
Education level ≥ college (%)	76.6	68.0	71.0	73.6	0.626
Occupation (desk work; %)	45.9	43.9	39.9	50.8	0.439
Metabolic syndrome (%)	28.3	27.5	33.8	29.0	0.493
Depression (%)	14.1	14.5	11.5	13.8	0.661
hsCRP (mg/L)	0.1 (0.7, 1.4)	1.1 (0.7, 1.4)	1.1 (0.8, 1.5)	0.6 (0.3, 1.0)	0.172
Hypnotic drug use (%)	4.8	3.3	2.2	3.0	0.173

^a^*BMI* body mass index, *PA* physical activity, *hsCRP* high-sensitivity C-reactive protein

^b^Obtained using analysis of variance for continuous variables and *χ*^2^ test for proportional variables

^c^Mean (95 % CI) (all such values)

The relationships between categories of isoflavone intake and sleep duration are shown in Table 2. After adjusting for age, sex, and BMI in Model 1, the odds ratios (ORs) for sleep duration increased across categories of isoflavone intake. The ORs for sleep duration across isoflavone intake categories Q1, Q2, Q3, and Q4 were 1.00 (reference), 0.93 (95 % CI, 0.54–1.61), 1.12 (95 % CI, 0.66–1.92), and 1.64 (95 % CI, 0.99–2.74), respectively (*p* for trend = 0.033). After adjusting for dietary intake in Model 2, the ORs for sleep duration across isoflavone intake categories Q1, Q2, Q3, and Q4 were 1.00 (reference), 0.98 (95 % CI, 0.56–1.70), 1.24 (95 % CI, 0.72–2.14) and 1.92 (95 % CI, 1.13–3.27), respectively (*p* for trend = 0.008). A similar association was observed between sleep duration and isoflavone intake in Model 3 (*p* for trend = 0.013).

**Table 2:** Adjusted relationship between daily intake of isoflavone and sleep duration

	Categories of isoflavone intake				*p* for trend^a^
	Q1 (Low)	Q2	Q3	Q4 (High)	
No. of subjects	269	269	269	269	
Sleep duration of 7–8 h/day (No.)	29	29	34	51	
Model 1^b^	1.00	0.93 (0.54, 1.61)^c^	1.12 (0.66, 1.92)	1.64 (0.99, 2.74)	0.033
Model 2^d^	1.00	0.98 (0.56, 1.70)	1.24 (0.72, 2.14)	1.92 (1.13, 3.27)^e^	0.008
Model 3^f^	1.00	0.94 (0.55, 1.65)	1.28 (0.73, 2.24)	1.84 (1.06, 3.18)^e^	0.013

^a^Obtained by multiple logistic regression analysis

^b^Adjusted for age, sex, and body mass index

^c^Adjusted odds ratio (95 % CI) (all such values)

^d^Further adjusted for intake of vitamins B12, C, and D; and coffee consumption

^e^Significantly different than category Q1, *p* < 0.05 (Bonferroni corrected)

^f^Further adjusted for smoking, drinking habits, physical activity, depressive symptoms, metabolic syndrome; high-sensitivity C-reactive protein level, education level, occupation, and hypnotic drug use

The relationships between categories of isoflavone intake and sleep quality are shown in Table 3. After adjusting for age, sex, and BMI in Model 1, the ORs for sleep quality across isoflavone intake categories Q1, Q2, Q3, and Q4 were 1.00 (reference), 1.33 (95 % CI, 0.94–1.87), 1.52 (95 % CI, 1.07–2.14), and 1.91 (95 % CI, 1.34–2.72), respectively (*p* for trend = 0.000). In Model 2, the ORs for sleep quality across isoflavone intake categories Q1, Q2, Q3, and Q4 were 1.00 (reference), 1.31 (95 % CI, 0.93–1.86), 1.49 (95 % CI, 1.05–2.12), and 1.85 (95 % CI, 1.28–2.67), respectively (*p* for trend = 0.001). A similar association was observed between sleep duration and isoflavone intake in Model 3 (*p* for trend = 0.002). The prevalence of either 7–8 h of sleep duration or better sleep quality was higher for subjects who consumed 26.74–83.06 mg/1000 kcal/d isoflavone (Q4) than for those who consumed 0–10.96 mg/1000 kcal/d isoflavone (Q1) in all models except Model 1 of the relationship between isoflavone intake and sleep duration (Bonferroni corrected *p* < 0.05). In addition, there was no significant interaction between isoflavone intake and sex, either for sleep duration or for sleep quality (*p* for interaction = 0.74 and 0.36, respectively). Likewise, there was no significant interaction between isoflavone intake and other confounding variables in the final models.

**Table 3:** Adjusted relationship between daily intake of isoflavone and sleep quality

	Categories of isoflavone intake				*p* for trend^a^
	Q1 (Low)	Q2	Q3	Q4 (High)	
No. of subjects	269	269	269	269	
No. with sufficient sleep	126	148	157	174	
Model 1^b^	1.00	1.33 (0.94, 1.87)^c^	1.52 (1.07, 2.14)^d^	1.91 (1.34, 2.72)^d^	<0.001
Model 2^e^	1.00	1.31 (0.93, 1.86)	1.49 (1.05, 2.12)^d^	1.85 (1.28, 2.67)^d^	0.001
Model 3^f^	1.00	1.30 (0.91, 1.84)	1.48 (1.03, 2.12)^d^	1.78 (1.22, 2.60)^d^	0.002

^a^Obtained by multiple logistic regression analysis

^b^Adjusted for age, sex, and body mass index

^c^Adjusted odds ratio (95 % CI) (all such values)

^d^Significantly different than category Q1, *p* < 0.05 (Bonferroni corrected)

^e^Further adjusted for intake of vitamins B12, C, and D; and coffee consumption

^f^Further adjusted for smoking, drinking habits, physical activity, depressive symptoms, metabolic syndrome, high-sensitivity C-reactive protein level, education level, occupation, and hypnotic drug use

## Discussion

The present study examined the relationships between daily isoflavone intake and sleep duration and quality. Our results suggest that high daily isoflavone intake from food is significantly related to optimal sleep duration (7–8 h) and better sleep quality. In addition, this relationship was not changed when adjusted for a number of potentially confounding variables, such as age, sex, BMI, total energy intake, coffee or vitamin consumption, smoking and drinking habits, level of education, occupation, depression, hsCRP level, or hypnotic drug use.

A previous study of 169 postmenopausal women showed that isoflavone treatment was effective in improving quality of life and decreasing climacteric symptoms, including sleep disturbance [21]. In addition, a randomized controlled trial study on postmenopausal women with insomnia indicated that sleep efficiency was significantly increased in the isoflavone treatment group compared to the placebo group [20]. These findings are consistent with those of the current study, which demonstrated a positive relationship between isoflavone intake and sleep quality. This consistency also suggests that there is a positive relationship between isoflavone intake and sleep quality, not only in postmenopausal women, but also in the general population.

Although the biological mechanisms involved in the relationship between isoflavone and sleep are unknown, there are some possible explanations. First, it has been determined that estrogen may affect some neurotransmitters in the brain, such as serotonin [28]. Serotonin is an important neurotransmitter, one function of which is to regulate the sleep-wake cycle [29]. Thus, it is possible that isoflavone acts as an estrogen mimetic to affect serotonergic function and regulate the sleep-wake cycle. Second, previous studies indicate that estrogen replacement therapy improves sleep quality [14, 30]. It is conceivable that isoflavones may also have beneficial effects on sleep quality in a similar way to estrogen, due to the estrogenic effects of isoflavone. Additionally, it has been shown that isoflavone intake has beneficial effects on cognitive function [31, 32] and endocrine such as IGF-1 [33]. Since cognitive function is positively related with sleep quality [34], and high plasma IGF-1 level is associated with increased delta sleep [35], we considered that isoflavone might also affect sleep by cognitive function or IGF-1. In addition, a previous study indicated that soy isoflavone supplementation decreased levels of oxidative stress in both men and women aged 22-56 years [36]. Further, oxidative stress may be a risk factor for poor sleep quality because an interventional study showed that anti-oxidant intake can improve the quality of sleep in sleep apnea syndrome patients [37]. Hence, we considered that antioxidant effects of isoflavone may also be a possible mechanism underlying the association between isoflavone intake and sleep status in present study.

Some limitations of this study should be discussed. First, our study does not exclude the possibility that isoflavone intake would not be associated with sleep quality and duration in premenopausal women who have higher level of estrogen. However, we could not examine the association of isoflavone intake with sleep duration and quality in premenopausal women because of the insufficient sample size. Although statistical adjustment for sex in the multivariate analysis showed a general association of isoflavone intake and sleep duration and quality, further study is necessary to examine whether the weak estrogen mimetic action of isoflavone could modify or improve the sleep duration and quality in premenopausal women. Second, because this study was a cross-sectional study, we could not conclude whether high isoflavone intake improved sleep duration and sleep quality, or whether sleep duration and sleep quality led to an increase in isoflavone intake. Third, we collected dietary data using a self-administered diet history questionnaire. Although this questionnaire has been validated and energy-adjusted values of dietary intake were used to minimize the effect of measurement errors derived from self-reported dietary data, real dietary habits were not observed. Finally, we adjusted for a number of confounding factors, but we cannot exclude the possibility that sleep duration and sleep quality are affected by other dietary habits that correlate with the habitual intake of isoflavone.

## Conclusions

In the present study, higher daily isoflavone intake from food was significantly associated with better sleep duration and higher sleep quality in a Japanese population, even after adjusting for potentially confounding factors. This suggests that the daily intake of isoflavones in food may have a potentially beneficial effect on sleep duration and quality, Prospective or interventional studies are required to clarify the causality.

## Abbreviations

BDHQ: Brief Self-administered Diet History Questionnaire
BMI: body mass index
CI: confidence intervals
hsCRP: High-Sensitivity C-Reactive Protein
IPAQ: International Physical Activity Questionnaire
MET: metabolic equivalent
PA: physical activity
SDS: Self-rating Depression Scale
